# Supplementary material for: Outcome of infants with bronchopulmonary dysplasia
Source: PLoS One. 2025 Nov 20;20(11):e0337083. doi: 10.1371/journal.pone.0337083 (PMC12633949; doi:10.1371/journal.pone.0337083)
Supplement: S1 File — (PDF) [file pone.0337083.s001.pdf]

Birthweight	Gender	Surfacta nt	Congenital Anomaly	Chorioa mnionitis	Congenital Heart Disease	Neonatal Sepsis	Mortality
876	Female	No	No	No	No	Yes	Alive
2220	Female	No	Yes	No	Yes	Yes	Alive
1044	Male	No	No	No	No	No	Alive
1750	Male	No	Yes	No	No	No	Alive
1100	Female	Yes	No	No	No	Yes	Dead
600	Female	No	No	No	No	No	Dead
986	Male	No	No	No	No	Yes	Dead
2228	Male	No	Yes	No	No	No	Dead
3684	Male	No	No	No	Yes	No	Dead
1080	Female	No	No	No	Yes	Yes	Dead
1000	Male	No	No	No	No	Yes	Dead
1380	Male	No	No	No	No	Yes	Alive
1100	Male	No	No	No	Yes	Yes	Alive
1000	Female	No	No	No	Yes	Yes	Dead
2210	Female	No	No	No	No	Yes	Dead
2436	Male	No	No	No	Yes	No	Alive
1160	Female	No	No	No	No	Yes	Alive
1876	Female	No	No	No	No	No	Dead
1600	Male	No	No	No	Yes	Yes	Dead
1102	Male	No	No	No	No	Yes	Dead
1088	Male	No	Yes	No	Yes	Yes	Dead
1000	Male	No	No	No	Yes	Yes	Alive
940	Male	Yes	No	No	Yes	Yes	Alive
1260	Male	Yes	No	No	No	Yes	Dead
880	Male	Yes	No	No	No	Yes	Dead
764	Female	No	No	No	No	Yes	Alive
1040	Female	No	No	No	No	No	Dead
2080	Female	No	No	No	No	Yes	Dead
1600	Female	No	No	No	Yes	Yes	Alive
1230	Male	No	No	No	No	Yes	Alive
1272	Female	No	No	No	Yes	Yes	Alive
1050	Female	Yes	No	No	No	No	Alive
1100	Male	Yes	No	No	Yes	No	Alive
1500	Male	Yes	No	No	Yes	Yes	Alive

1468	Female	No	No	No	Yes	Yes	Alive
800	Female	No	No	No	Yes	No	Dead
1560	Male	No	Yes	No	Yes	No	Alive
1072	Male	Yes	No	No	Yes	Yes	Dead
1866	Male	No	No	No	No	No	Dead
1100	Female	No	No	No	No	Yes	Alive
985	Female	No	No	No	No	Yes	Dead
950	Male	No	No	No	Yes	Yes	Dead
3506	Male	Yes	No	No	Yes	Yes	Alive
1900	Male	No	Yes	No	No	Yes	Dead
965	Female	No	No	No	Yes	No	Alive
1300	Male	No	No	No	No	Yes	Alive
1190	Male	No	No	No	Yes	Yes	Alive
1195	Male	Yes	No	No	No	Yes	Dead
1900	Male	No	Yes	No	Yes	No	Alive
1000	Male	No	No	No	Yes	No	Alive
1218	Female	No	No	No	No	No	Alive
938	Female	No	No	No	No	Yes	Dead
1450	Female	No	No	No	No	No	Dead
760	Male	No	No	No	No	Yes	Alive
1300	Female	No	No	No	No	No	Dead
1360	Female	No	No	No	No	No	Alive
870	Female	No	No	No	No	No	Dead
2460	Male	No	No	No	No	No	Dead
1319	Female	No	No	No	No	Yes	Dead
940	Female	Yes	No	No	Yes	Yes	Dead
1334	Male	Yes	No	No	No	Yes	Dead
900	Male	No	No	No	Yes	No	Dead
990	Male	No	No	No	No	Yes	Alive
909	Male	No	No	No	No	No	Alive
2700	Female	No	No	No	No	Yes	Dead
900	Female	No	No	No	Yes	Yes	Alive
1300	Female	No	No	No	No	Yes	Alive
1184	Male	Yes	No	No	No	Yes	Dead
960	Female	No	No	No	Yes	Yes	Alive
1148	Female	Yes	No	No	No	No	Alive
1350	Female	No	No	Yes	Yes	Yes	Dead
1850	Male	No	No	No	Yes	Yes	Dead

1120	Male	No	No	No	No	No	Alive
2597	Male	No	No	No	No	Yes	Alive
1050	Male	No	No	No	Yes	Yes	Alive
1200	Male	Yes	No	No	No	Yes	Dead
1250	Female	No	No	No	Yes	Yes	Dead
1100	Female	No	No	No	Yes	No	Alive
1200	Female	No	No	No	No	Yes	Alive
1500	Female	No	No	No	Yes	No	Alive
1160	Female	No	No	No	No	Yes	Alive
1240	Male	Yes	No	No	Yes	No	Alive
850	Female	No	No	No	No	Yes	Dead
2902	Male	No	No	No	No	Yes	Dead
3550	Male	No	No	No	No	No	Alive

Oxygen Therapy	Mechanical Ventilator	CPAP	Oxygen Supplementation	Small for Gestational Age	Length of Stay (day)	Hospitalization Frequency	BPD Severity
No	No	No	No	Yes	69	1	2
Yes	Yes	No	Yes	No	45	1	1
Yes	Yes	No	Yes	No	50	1	1
Yes	No	No	Yes	No	47	1	2
Yes	Yes	No	No	Yes	9	1	2
Yes	Yes	Yes	No	No	26	2	2
No	No	No	No	No	77	1	3
No	No	No	No	Yes	10	2	2
Yes	Yes	Yes	No	No	108	1	1
Yes	No	Yes	No	Yes	73	1	3
Yes	Yes	No	No	No	26	1	1
No	No	No	No	No	49	1	1
No	No	No	No	No	96	1	3
Yes	Yes	No	No	Yes	59	1	3
Yes	Yes	Yes	No	Yes	40	1	3
No	No	No	No	No	63	1	2
Yes	No	Yes	No	No	37	1	1
Yes	Yes	Yes	Yes	No	86	1	1
Yes	Yes	No	No	No	66	3	3
No	No	No	No	No	49	2	1
Yes	Yes	Yes	No	No	192	1	2
Yes	Yes	No	No	Yes	75	1	3
Yes	Yes	Yes	Yes	No	74	1	1
Yes	No	Yes	No	No	81	1	3
No	No	No	No	No	13	2	3
No	No	No	No	No	69	1	1
Yes	Yes	Yes	No	No	100	1	2
Yes	Yes	Yes	No	No	188	1	3
No	No	No	No	No	50	1	2
Yes	No	Yes	No	No	44	1	3
No	No	No	No	No	44	1	3
Yes	No	Yes	Yes	No	46	1	2
Yes	Yes	No	No	No	44	1	2
Yes	Yes	Yes	No	No	76	1	1

No	No	No	No	No	39	1	1
Yes	Yes	Yes	No	No	127	1	3
Yes	Yes	No	No	No	60	1	1
Yes	Yes	Yes	No	No	93	1	3
No	No	No	No	No	10	1	2
No	No	No	No	No	52	1	3
Yes	Yes	Yes	Yes	No	53	1	1
Yes	Yes	No	No	No	15	2	3
Yes	Yes	No	No	No	24	1	1
Yes	Yes	Yes	Yes	Yes	57	1	3
Yes	Yes	Yes	Yes	No	10	1	3
Yes	No	Yes	No	No	22	1	3
Yes	Yes	Yes	Yes	No	60	1	1
No	No	No	No	No	18	1	1
Yes	No	Yes	Yes	Yes	92	1	2
Yes	Yes	Yes	No	No	80	1	2
No	No	No	No	Yes	36	1	2
Yes	No	Yes	No	No	104	2	2
Yes	Yes	No	No	No	15	1	2
No	No	No	No	Yes	54	1	2
No	No	No	No	Yes	49	1	1
No	No	No	No	Yes	37	1	1
Yes	Yes	Yes	Yes	Yes	30	1	1
Yes	Yes	Yes	No	No	7	2	1
Yes	Yes	Yes	No	No	33	1	1
Yes	Yes	No	No	No	40	1	2
Yes	No	Yes	No	Yes	68	1	1
Yes	Yes	Yes	Yes	No	89	1	1
Yes	Yes	Yes	Yes	No	79	1	1
Yes	Yes	Yes	No	No	123	1	3
Yes	Yes	No	No	No	87	1	3
Yes	No	Yes	No	Yes	62	1	2
Yes	Yes	Yes	Yes	No	40	1	1
Yes	Yes	Yes	No	No	79	2	2
Yes	Yes	Yes	Yes	Yes	101	1	1
Yes	No	Yes	No	No	57	1	3
Yes	Yes	Yes	Yes	Yes	66	1	1
Yes	Yes	Yes	Yes	Yes	72	1	1

Yes	Yes	Yes	Yes	No	57	1	1
Yes	No	Yes	No	No	33	1	2
Yes	Yes	Yes	Yes	No	103	1	3
Yes	Yes	No	No	No	26	1	3
Yes	Yes	Yes	Yes	No	44	1	2
Yes	Yes	Yes	Yes	No	25	1	2
Yes	Yes	Yes	Yes	No	26	1	1
Yes	No	Yes	Yes	Yes	34	1	2
Yes	Yes	Yes	No	No	65	1	2
Yes	Yes	Yes	No	No	52	1	2
Yes	Yes	No	No	Yes	4	1	3
Yes	Yes	No	No	No	35	1	1
Yes	Yes	Yes	Yes	No	33	2	3
